# Increased fat mass and obesity risk after elexacaftor–tezacaftor–ivacaftor therapy in young adults with cystic fibrosis

**DOI:** 10.3389/fnut.2024.1477674

**Published:** 2024-11-07

**Authors:** Ana Merino Sánchez-Cañete, Concepción Marina López Cárdenes, Saioa Vicente Santamaría, José Ramón Gutiérrez Martínez, Marta Suárez González, María Álvarez Merino, David González Jiménez

**Affiliations:** ^1^Cystic Fibrosis Unit, Department of Pediatrics, University Hospital Ramón y Cajal, Madrid, Spain; ^2^Cystic Fibrosis Unit, University Hospital Central de Asturias, Oviedo, Spain

**Keywords:** cystic fibrosis, body composition, nutritional status, bioelectrical impedance, CFTR modulators

## Abstract

**Background:**

When people with cystic fibrosis (PwCFs) are treated with cystic fibrosis transmembrane conductance regulator protein modulator (CFTRm), it leads to changes in body composition. An easy, non-invasive, and reproducible method to assess this is by means of electrical bioimpedance measurement, which complements the information provided by the already-known anthropometric parameters.

**Methods:**

Seventeen adults with cystic fibrosis in treatment with elexacaftor–tezacaftor–ivacaftor (ETI) were recruited for a prospective, longitudinal, single-center study over 12 months. Study variables included weight, body mass index (BMI), and body composition by bioelectrical impedance analysis (BIA) [including fat mass (FM) and fat-free mass (FFM)].

**Results:**

At 12 months, there was an increase in overweight patients (5.9% vs. 23.5%) and a statistically significant increase in BMI at 6 and 12 months. An increase in FM and FFM was observed at 12 months. The increase was greater in FM (95% CI) from a baseline of 1.77% (0.00–3.54; *p* = 0.050) at 6 months and 2.64% (0.48–4.81; *p* = 0.020) at 12 months.

**Conclusion:**

After treatment with ETI, there was an increase in weight, BMI, and FM more than FFM in PwCF. These changes would be confirmed in long-term studies to improve nutritional management.

## 1 Introduction

Since European Medicines Agency (EMA) approved elexacaftor–tezacaftor–ivacaftor (ETI) for people with cystic fibrosis (PwCF) in 2020, the improvement in lung function and the decrease in sweat chloride have been superior to that demonstrated in PwCF treated with previous generations of cystic fibrosis transmembrane conductance regulator protein modulator (CFTRm) (1–3).

Improvements in anthropometric and body composition changes appear to be observed (1, 4–6) regarding a special interest as a better nutritional status is associated with improved quality of life, survival (7), and lung function in cystic fibrosis (*CF*) (5, 8).

In recent years, the nutritional status of patients has been defined by anthropometric parameters such as weight, height, and body mass index (BMI). Using new methods, such as bioelectrical impedance analysis (BIA), allows us to complete the information on body composition in a non-invasive, valid, and safe way for PwCF (9, 10).

In our study, we analyzed the anthropometric and body composition changes observed in a cohort of PwCF over 18 years of age with *CF* and undergoing treatment with ETI.

## 2 Methods

### 2.1 Study design and outcome variables

We designed a prospective, longitudinal study in a single *CF* Unit. PwCF data were collected before ETI initiation and at 6 and 12 months post-treatment. The clinical research ethics committee of the participating hospital approved the study. Informed consent was obtained from all subjects. We included adult patients who were 18 years or older. The mandatory requirements were that all the participants were diagnosed with *CF*, and the genotype had at least one F508del mutation. All participants initiated triple therapy with ETI at the time of the study, regardless of prior treatment with CFTRm. The exclusion criteria were patients with only baseline bioimpedance measurement without subsequent follow-up and patients with incomplete data and PwCF who had to stop treatment at some point during the study.

### 2.2 Anthropometric measures

Regarding the anthropometric parameters used, they were classified into four groups according to their BMI according to WHO guidelines: underweight (<18.5 kg/m^2^), adequate weight (18.5–24.9 kg/m^2^), overweight (25–29.9 kg/m^2^), or obese (≥30 kg/m^2^). In addition, we follow the recommendations of the Cystic Fibrosis Foundation and according to The European Society for Clinical Nutrition and Metabolism (ESPEN) guidelines 2024 for patients with *CF*: Women maintain a BMI of at least 22 kg/m^2^ and men a BMI of at least 23 kg/m^2^.

### 2.3 Body composition assessment

Body composition was studied with a BIA AKERN 101 device (Akern, Montachiello, Pisa, Italy). The study was carried out with the subjects lying supine on a stretcher with their arms separated from the trunk by 30° and their legs separated by 45°. Four electrodes (Biatrodes, Akern) were placed on the extremities, two on the right hand and another two on the right foot, with a 4–5-cm space between them. These were connected by wire to the device, thus providing the resistance and reactance values. These data, together with the subject’s weight and height, age, sex, ethnicity, and physical activity, were entered into the device’s accompanying software (BODYGRAM PLUS, Akern), and the values of FM, FFM, and total body water (TBW) were recorded.

### 2.4 Statistical analysis

Study data were collected and managed using REDCap (11) electronic data capture tools hosted at Sociedad Española de Gastroenterología, Hepatología y Nutrición Pediátrica (SEGHNP).^1^ The technical support was made by the AEG REDCap Support Unit, shared with Asociación Española de Gastroenterología (AEG).

Descriptive statistics were performed on demographic, clinical, and body composition data. Shapiro–Wilk test was used to assess normality. Paired Student’s *t*-test was used to compare pre- and post-ETI values for all variables where the normality assumption was met. Statistical analysis was performed with STATA software, version 13.1. A statistically significant result was considered to be *p* < 0.05.

## 3 Results

### 3.1 Participant baseline characteristics

Finally, seventeen patients with baseline body impedance values and at least one other at 6 or 12 months were recruited. The ages were between 20 and 47 years, with a mean of 32.2 years old, without differences in sex. More than half of the participants (55.9%) were homozygous for the F508del mutation, and 88.2% were on enzyme replacement therapy (ERT) for pancreatic insufficiency.

### 3.2 Anthropometric and body composition parameters

During follow-up, patients presented a statistically significant increase in mean BMI (paired Student’s *t*-test): 0–6 months 0.77 (CI 95%: 0.19–1.36) kg/m^2^ (*p* = 0.013); 6–12 months 0.59 (CI 95%: 0.13–0.92) kg/m^2^ (*p* = 0.013); and 0–12 months 1.29 (CI 95%: 0.56–2.04) kg/m^2^ (*p* = 0.002).

According to WHO BMI guidelines, there was an increase in overweight (25–29.9 kg/m^2^) patients after 12 months on ETI (*p* = 0.083, McNemar’s test), being 5.9% (1/17 patients) of patients at baseline vs. 23.5% (4/17 patients) after 12 months of study.

Regarding the BMI nutritional target according to the *CF* Foundation and ESPEN 2024 guidelines, two patients reached the BMI recommendations for improving respiratory parameters (FEV1): At baseline, 8 patients (41%) had a BMI of at least 22 in women and 23 in men, and at 12 months of treatment, 10 patients had at least this BMI.

There was a significant increase in patient weight after 12 months of ETI (60.5 ± 9.3 vs. 64.20 ± 10.88, *p* = 0.001, paired Student’s *t*-test) and a significant increase in FM (kg) in the same interval (9.51 ± 4.46 vs. 12.00 ± 4.83, *p* = 0.006, paired Student’s *t*-test).

Table 1 summarizes the main data with the changes in body impedance.

**Table 1:** Anthropometric and body composition parameters.

	0–6 months				6–12 months				0–12 months			
	*N*	Baseline	6 months	p**	*N*	6 months	12 months	p**	*N*	Baseline	12 months	p**
Weight (kg)	17	60.5 ± 9.30	62.8 ± 10.12	<0.01	17	62.8 ± 10.12	64.2 ± 0.88	0.024	17	60.5 ± 9.30	64.2 ± 10.88	0.001
BMI (kg/m^2^)	17	22.3 ± 1.81	23.0 ± 1.61	0.013	17	23.0 ± 1.61	23.6 ± 1.93	0.012	17	22.3 ± 1.81	23.6 ± 1.93	0.001
FM												
FM (kg)	17	9.34 ± 4.37	10.91 ± 4.70	0.021	16	10.87 ± 4.85	12.00 ± 4.83	0.071	16	9.51 ± 4.46	12.00 ± 4.83	0.006
Body fat (%)	17	15.88 ± 7.39	17.65 ± 7.20	0.050	16	17.39 ± 7.36	18.64 ± 7.13	0.200	16	16.00 ± 7.62	18.64 ± 7.13	0.020
FMI (kg/m^2^)	17	3.49 ± 1.62	4.04 ± 1.62	0.022	16	4.01 ± 1.67	4.43 ± 1.75	0.092	16	3.54 ± 1.66	4.43 ± 1.75	0.007
FFM												
FFM (kg)	17	50.89 ± 10.40	52.04 ± 11.20	0.015	16	52.69 ± 11.23	50.73 ± 17.76	0.663	16	51.53 ± 10.40	50.74 ± 17.76	0.851
Body fat free (%)	17	84.12 ± 7.39	82.35 ± 7.20	0.050	16	82.61 ± 7.36	81.36 ± 7.13	0.200	16	84.00 ± 7.62	81.36 ± 7.13	0.020
FMMI (kg/m^2^)	17	18.59 ± 2.19	19.05 ± 2.44	0.007	16	19.21 ± 2.42	19.31 ± 2.24	0.654	16	18.75 ± 2.17	19.31 ± 2.24	0.003
Body cell mass												
BCMI (kg/m^2^)	17	10.72 ± 1.84	10.95 ± 2.13	0.136	16	11.06 ± 2.14	11.16 ± 2.00	0.621	16	10.84 ± 1.83	11.16 ± 2.00	0.045
Body cell mass (%)	17	29.35 ± 7.14	29.94 ± 8.09	0.154	16	30.39 ± 8.13	30.72 ± 7.88	0.539	16	29.79 ± 7.13	30.72 ± 7.88	0.050
TBW												
TBW (%)	17	61.18 ± 5.37	61.31 ± 7.27	0.930	16	61.58 ± 7.43	59.31 ± 5.08	0.102	16	61.08 ± 5.53	59.31 ± 5.08	0.035

BMI, body mass index; FM, fat mass; FFM, fat-free mass; TBW, total body water; BCMI, body cell mass index; *Values are expressed as mean ±DS; ** Paired T Student test.

**Figure 1:**
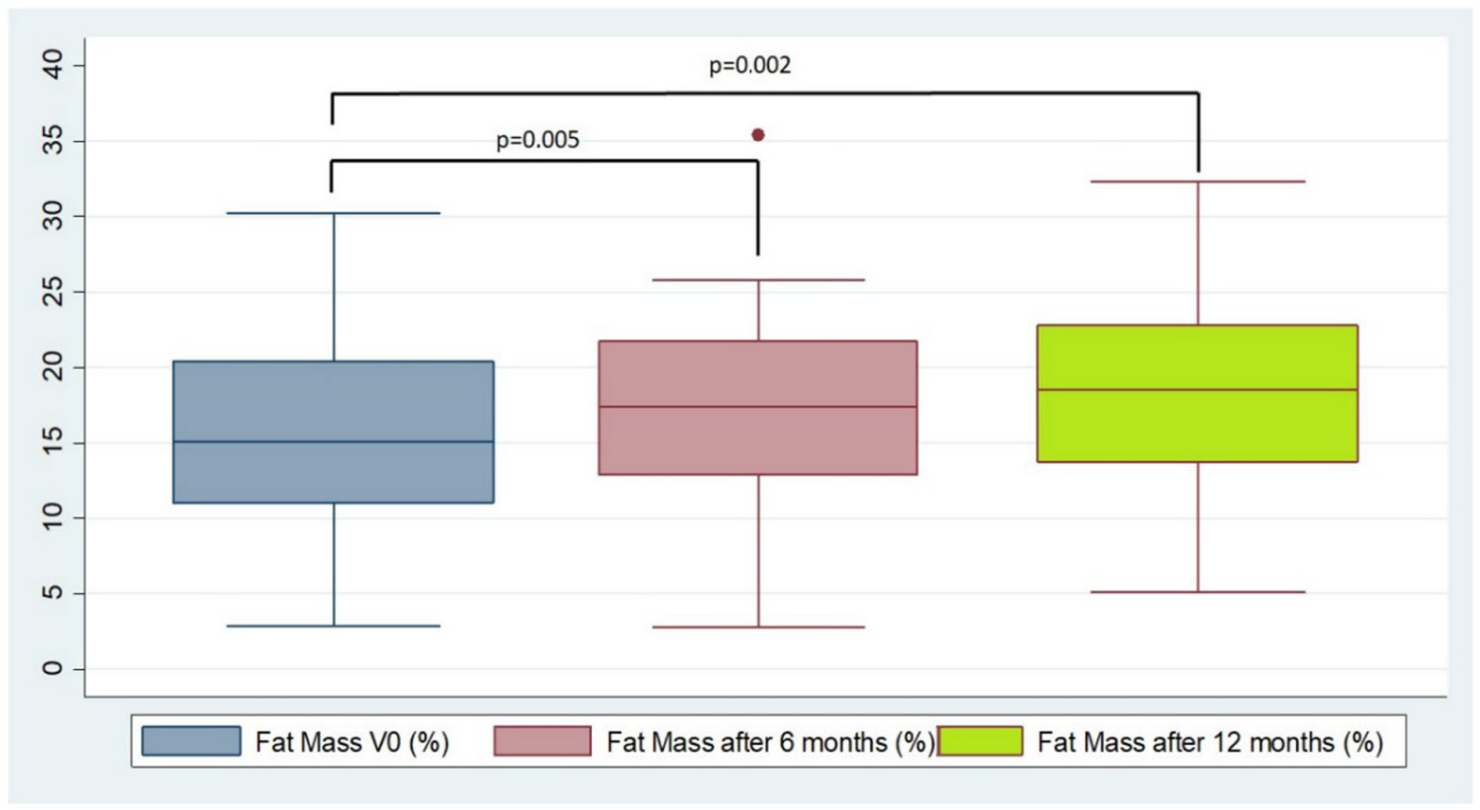
Percentage of fat mass (FM) at baseline at 6 months and 12 months.

We observed an increase in total FM and FFM compared with basal values; however, in proportion, there is a greater increase in the percentage of FM compared to FFM (paired Student’s *t*-test): Mean 1.77 (IC 95%: 0.00–3.54; *p* = 0.050) between 0 and 6 months and 2.64 (IC 95%: 0.48–4.81; *p* = 0.020) between 0 and 12 months (Figure 1). We do not find differences in FM and FFM percentages between 6 and 12 months. Figure 1 shows the changes in FM at baseline, 6 months, and 12 months of the study.

## 4 Discussion

Observational studies (1, 4) have already shown improvements in lung function and reductions of sweat chloride but also a significant change in body composition (5–7).

In this context, there is growing interest in body composition changes that occur after the initiation of ETI, which have been increasing (12). A study by King et al. already evidenced these changes in a cohort of adults on Ivacaftor treatment (1). In addition, if we look at the German Cystic Fibrosis Registry 2022 (13), we can see a change in the trend in the BMI of patients. In this registry, an increase in the percentage of patients reaching the optimal BMI (women 22–23.9 kg/m^2^ and men 23.0–24.9 kg/m^2^) can be observed between 2010 and 2022, at the time of the start of CFTR modulators (13). As in our study, an increase in BMI, defined as overweight by WHO (14), was observed in the age groups of patients included in our study and between the years corresponding to the start of CFTR modulators.

However, to our knowledge, there are currently no studies using BIA to assess body composition in PwCF on ETI. Our study observed an increase in weight *Z*-score and BMI. Both were significant at 6 and 12 months of treatment. Of note is the increase in overweight patients with possible short- and long-term consequences. Similarly, studies such as that of Granados et al. also show a significant increase in weight (7, 15).

However, a BMI in the appropriate range according to reference values does not necessarily imply an adequate body composition (8, 10, 16–18). This is the case in Engelen et al. where BMI could not estimate FFM depletion in more than half of the patients. Furthermore, if not well matched to body composition, the appropriate BMI value in *CF* is constantly changing (15, 17–20). In this respect, our results support the conclusion of similar studies. If we compare these values with those of the population in the study by King et al., the population has a similar BMI (22.3 vs. 23.3 k/m^2^); however, our sample has a lower percentage of FM (15.9 vs. 23.6) and a higher percentage of FFM (84.1 vs. 76.4).

Therefore, it is important to highlight patients who, despite having a normal weight (kg), have a BMI (kg/m2) in the obese range with a high percentage of FM and low FFM as it is related to worse lung function. In percent when compared to overweight and obese PwCF (18).

Therefore, we consider that this study can be a reference point for studying body composition by this method in PwCF treated with ETI.

Regarding body composition, several studies have shown changes in body compartments (15, 19, 20). There appears to be a tendency for rapid weight and BMI gain during the first months of therapy. These initial changes and subsequent stabilization have been specifically studied with the onset of ETI and assessed with different methods, such as dual-energy X-ray absorptiometry (DXA) (7). It is of interest to extend studies explaining this rapid initial improvement and to assess the stabilization of anthropometric parameters and their relationship with changes in lung capacity. Both in our study and in King et al., there was a significant increase in weight at the expense of FM in the first 6 months. However, after 2 years of follow-up in the ivacaftor-treated cohort, weight and FM have attenuated with the stabilization of FFM (1). In our study, following the cohort for a shorter period (12 months), weight and FM continue to increase significantly, with a slight depletion of FFM.

The increase in weight and the fat component in the cohort of patients has been previously attempted to be explained in patients treated with ivacaftor (21, 22). However, it is not known whether this mechanism is exclusively due to CFTRm (23, 24).

There are studies assessing body composition changes after initiation of ETI using methods such as DXA or after using one or a combination of two CFTR modulators (1, 7). However, our study provides the first insight into the body composition behavior of patients using BIA in the first months of treatment with ETI. Sustained improvement in these results would be expected with continued treatment. However, these preliminary data and results must be confirmed over the next few months of treatment.

The study’s limitations are the small sample size and the lack of a control group. Furthermore, it is a short-term study, and the results obtained are early changes. In addition, factors that could influence body composition, such as physical activity and dietary intake, have not been included in the analysis. Finally, our body composition data are presented as percentages and kilograms, although the use of values for resistance and reactance is preferred by ESPEN guidelines 2024.

However, if larger studies confirm these results, it may lead to a change in the approach to these patients. It will be important to emphasize nutritional therapy, individualization, and monitoring of body composition from the start of therapy.

## 5 Conclusion

The initiation of treatment with CFTR modulators has led to a change in the management of *CF.* In general, anthropometric changes are reflected in weight gain, which in the first 6 months reflects an increase in both FM and FFM, both of which are significant. It would be interesting to analyze the evolution of these parameters in the long term to verify the stabilization of the parameters and improve the nutritional approach.

## Data Availability

The original contributions presented in the study are included in the article/supplementary material, further inquiries can be directed to the corresponding author.

## Footnotes

^1^ www.seghnp.org
